# Cancer Cells Upregulate Tau to Gain Resistance to DNA Damaging Agents

**DOI:** 10.3390/cancers15010116

**Published:** 2022-12-24

**Authors:** Thomas Rico, Marine Denechaud, Raphaelle Caillierez, Thomas Comptdaer, Eric Adriaenssens, Luc Buée, Bruno Lefebvre

**Affiliations:** 1 Alzheimer & Tauopathies, UMR-S1172, Lille Neuroscience & Cognition, CHU-Lille, Inserm, Univ. Lille, F-59000 Lille, France; 2 UMR 9020-U 1277—CANTHER—Cancer Heterogeneity Plasticity and Resistance to Therapies, CHU-Lille, Inserm, Univ. Lille, F-59000 Lille, France

**Keywords:** Tau, microtubules, double strand DNA breaks, resistance, breast cancer cells, cNHEJ, 53BP1

## Abstract

**Simple Summary:**

The role of Tau in genome protection and/or repair in neurons suggests that Tau expression in cancer cells could be involved in resistance to conventional anti-cancer treatments, in particular those inducing DNA damage. Knockdown of Tau in breast cancer cell lines improved the cellular response and resulted in a significant decrease of mouse-xenograft breast tumor volume after DNA damaging agent treatments by impairing the classical nonhomologous end-joining pathway. Tau allows 53BP1 to translocate to the nucleus in response to DNA damage by chaperoning microtubule protein trafficking.

**Abstract:**

Recent reports suggested a role for microtubules in double-strand-DNA break repair. We herein investigated the role of the microtubule-associated protein Tau in radio- and chemotherapy. Noticeably, a lowered expression of Tau in breast cancer cell lines resulted in a significant decrease in mouse-xenograft breast tumor volume after doxorubicin or X-ray treatments. Furthermore, the knockdown of Tau impaired the classical nonhomologous end-joining pathway and led to an improved cellular response to both bleomycin and X-rays. Investigating the mechanism of Tau’s protective effect, we found that one of the main mediators of response to double-stranded breaks in DNA, the tumor suppressor p53-binding protein 1 (53BP1), is sequestered in the cytoplasm as a consequence of Tau downregulation. We demonstrated that Tau allows 53BP1 to translocate to the nucleus in response to DNA damage by chaperoning microtubule protein trafficking. Moreover, Tau knockdown chemo-sensitized cancer cells to drugs forming DNA adducts, such as cisplatin and oxaliplatin, and further suggested a general role of Tau in regulating the nuclear trafficking of DNA repair proteins. Altogether, these results suggest that Tau expression in cancer cells may be of interest as a molecular marker for response to DNA-damaging anti-cancer agents. Clinically targeting Tau could sensitize tumors to DNA-damaging treatments.

## 1. Introduction

Although defects in DNA damage response lead to genomic instability, this can also represent the “Achilles’s heel” of cancer cells. Deficiency in any specific repair pathway makes cells more dependent on the remaining DNA-repair pathways, rendering them more vulnerable to certain DNA-targeting therapies. Double-strand DNA breaks (DSBs) are considered the most lethal DNA lesions and are commonly induced in cancer radio- or chemotherapy. However, one of the main limitations of DSB-associated therapies is the tumor resistance developed before or after treatment [1,2]. Thus, it will be important to decipher the underlying molecular mechanism of this resistance to improve existing regimens.

In cells, DSBs activate the DNA damage response pathways through the kinases ATM, ATR, and DNA-PKcs, which in turn activate the proteins involved in two main pathways, classical nonhomologous end-joining (cNHEJ) and homologous recombination (HR). cNHEJ mediates repair by directly rejoining DSB ends, contrary to HR, which utilizes a homologous DNA sequence to guide DNA repair [3,4]. Which of the two repair routes is used depends in part on the cell cycle phase. Since HR relies on the presence of a sister chromatid, it can be effective only in the late S/G2 phase, while the NHEJ pathway is effective throughout the cell cycle. Recruitment of specific factors to DNA lesions is also essential to initiate DNA repair, and this is regulated, in part, by histone post-translational modifications [5]. In this context, 53BP1 and BRCA1 (breast cancer 1), together with auxiliary factors, play a pivotal role in the pathway choice. They trigger either cNHEJ or HR, respectively. 53BP1 blocks the DNA end resection necessary for HR. cNHEJ accounts for most DSB repair in mammalian cells [6,7,8].

DNA repair mechanisms are multifaced, but recent studies demonstrated an essential role for microtubules. First, microtubule dynamics were demonstrated to be crucial in the intracellular trafficking of DNA repair proteins [9]. Second, it has been demonstrated that the interaction between microtubules, linkers of the nucleoskeleton, and cytoskeleton’ complexes increase the mobility of DSBs toward repair complexes [10].

Tau was first described as a neuronal microtubule-associated protein (MAPT), and when it aggregates, it is a major player in neurodegenerative diseases like Alzheimer’s [11]. However, Tau has many other cellular functions [12]. In addition to microtubule dynamics, it interferes with several biological processes, such as signaling pathways and RNA metabolism, and it even contributes to the inflammatory response. Furthermore, Tau is not only found in the cytoplasm but also in the cell nucleus, where it participates in chromatin organization in neuronal and cancer cells [13,14]. Several data suggested a role for Tau in DNA repair [15]. In particular, Tau deletion induces an increase in DSBs and slower DNA repair in neurons after reactive oxygen species induction triggered by hyperthermia [16,17]. However, the molecular mechanism remains elusive. In this regard, it has been demonstrated in vitro that Tau protects DNA from free radical damage [18]. The current explanation of this protective effect is based on Tau’s ability to bind DNA in a sequence-independent manner [19]. Although Tau is known as a neuronal protein, a large body of literature has shown that its expression is increased in several types of cancer and may be associated with acquired resistance to Taxanes [20,21]. Tau binds in the same binding site as paclitaxel and consequently competes with this drug [22].

The role of Tau in genome protection and/or repair suggests that Tau expression in cancer cells could be involved in resistance to conventional anti-cancer treatment, in particular those inducing DNA damage. Starting from this hypothesis, we evaluated the role of Tau in DNA repair in breast cancer cell lines.

## 2. Materials and Methods

### 2.1. Materials and Plasmids

pcDNA3-Tau4R and Tau-GFP have been described elsewhere [14,23,24]. pimEJ5GFP was a gift from Jeremy Stark (Addgene plasmid # 44026) (Watertown, MA, USA) [25]. pDRGFP (Addgene plasmid # 26475) and pCBASceI (Addgene plasmid # 26477) were a gift from Maria Jasin [26]. Short hairpin Tau and RNA Ctrl vectors were purchased from Santacruz. Doxorubicin, cisplatin, oxaliplatin, 6-thioguanine, and hypoxanthine were purchased from Sigma-Aldrich (Saint-Louis, MO, USA), and bleomycin from Calbiochem (from Sigma, St. Louis, MO, USA). Cells were exposed to ionizing radiation (IR) using an X-ray machine (Clinac23X, Varian Medical Systems, Palo Alto, CA, USA).

### 2.2. Cell Culture and Transfection

HeLa cells, MCF7, and MDA-MB-231 were cultured in Dulbecco’s Modified Eagle’s Medium with 10% fetal bovine serum, 2 mM L-glutamine, and 50 U/mL penicillin/streptomycin (Gibco, Life Technologies, Carlsbad, CA, USA) at 37 °C in 5% CO_2_ humidified air. CHO-K1 cells were cultured in RPMI1640 (Gibco) with 10% fetal bovine serum, 2 mM L-glutamine, and 50 U/mL penicillin/streptomycin (Gibco). MCF7 shctrl and shTau cells have been described previously [14]. Transient and stable transfection experiments were performed using the lipofectamine LTX reagent according to the manufacturer’s guidelines (Invitrogen). To isolate stably transfected HeLa clones, cells were transfected with pDRGFP or pimEJ5GFP plasmids and selected with puromycin (2 μg/mL). Clones were isolated and tested for GFP expression after pCBASceI transfection. Of the six clones tested for GFP, one was chosen for further study. For CHO-K1 GFP or GFP-Tau, cells were transfected with plasmids encoding GFP or GFP-Tau and selected with G418 (200 μg/mL). Stably transfected cells were isolated using flow cytometry and cell sorting (Sony SH800, San Jose, CA, USA).

### 2.3. Cellular Extracts and Western Blotting

For the preparation of whole cell extracts, cells were washed twice in ice-cold PBS and scraped in lysis buffer [50 mM Tris-HCl (pH 7.5), 150 mM NaCl, 1 mM EDTA, 1 mM EGTA, 1% Triton X-100, and protease inhibitor mixture (Roche, Rotkreuz, Switzerland)]. The lysate was rotated for 1 h at 4 °C and centrifuged for 15 min at 4 °C. Nuclear and cytoplasmic extracts were prepared as described previously [24]. Briefly, cells were harvested, washed with PBS, and resuspended in buffer A (10 mM Hepes pH = 7.8, 10 mM KCl, 0.5 mM EDTA, 1 mM DTT, and complete protease inhibitors [Roche]). Nuclei were recovered by centrifugation for 10 min at 2000 g, resuspended in nuclear lysis buffer (50 mM Hepes pH 7.4, 1.5 mM MgCl2, 420 mM NaCl, 1 mM dithiothreitol [DTT], and complete protease inhibitors [Roche]), and then incubated for 1 h in a rotation wheel at 4 °C to extract nuclear proteins.

For western blotting, proteins were solubilized in an SDS loading buffer and analyzed by SDS-PAGE. Primary antibodies used in western blotting experiments were directed against Tau Cter [14], 53BP1 (Cell Signaling), H3 (Millipore), Hsp90 (Santa Cruz), and β-actin (Sigma, St Louis, MO, USA). Secondary antibodies coupled to HRP were from Sigma-Aldrich. Immune complexes were detected using the ECL+ system from Amersham/GE Healthcare and observed with an Image Reader LAS4000 (Fujifilm, Courbevoie, France). Quantification was performed by densitometry using ImageJ software. Original blots see Supplementary File S1.

### 2.4. Cell Fractionation into Cytosolic and Microtubule Fractions

Cell fractionation was performed as described previously [27]. Briefly, equal amounts of MCF7 shCtrl and shTau cells were recovered in buffer A (80 mM Pipes, pH 6.8, 1 mM MgCl_2_, 2 mM EGTA, 30% glycerol, 0.1% Triton X-100, and complete protease inhibitors [Roche]). After ultracentrifugation at 100,000× *g* at 21 °C for 18 min, supernatants were collected as cytosolic fractions. The pellets (microtubule fraction) were recovered in RIPA buffer and sonicated. Samples were mixed with LDS buffer, and equal volumes were loaded for SDS-PAGE and analyzed by immunoblotting.

### 2.5. Hprt Mutant Frequency Determination

*Hprt* mutant frequency was determined by measuring the clonogenicity of cells, as previously described [28]. Briefly, CHO-K1 cells stably transfected with GFP or GFP-Tau were grown in hypoxanthine medium for five days to eliminate preexisting *Hprt* mutants, then 1.5 × 10^6^ cells were plated in 75 cm^2^ flasks. After 24 h of culture, cells were or were not treated with cisplatin (10 µM, 2 h), oxaliplatin (20 µM, 2 h), or X-rays (2 Gy). Cells were maintained for eight days to allow the expression of the *Hprt* mutant phenotype. After this period, 2 × 10^5^ cells were plated in 100 mm petri dishes in a complete medium containing 6 µg/mL of 6-thioguanine for the selection of *Hprt* mutants. In parallel, 200 cells were seeded in 35 mm dishes (12 dishes/condition) with a non-selective medium. Ten days later, colonies formed in both selective and non-selective media were fixed, stained with 4% Giemsa, and scored. Colony efficiency was expressed as a ratio between the number of colonies and the number of seeded cells. Mutant frequency was expressed as a ratio between the colony efficiency of *Hprt* mutants and those cells cultured in a non-selective medium.

### 2.6. Immunofluorescence

Cells were fixed in 4% paraformaldehyde for 30 min at room temperature. Permeabilization was carried out in 0.2% Triton X-100 in phosphate-buffered saline for 10 min at room temperature. After 1 h of saturation in 2% bovine serum albumin, immunostaining was performed using γH2AX (Millipore, Burlington, MA, USA), 53BP1 (Cell Signaling, Danvers, MA, USA), and γ-tubulin (Millipore). These antibodies were revealed via secondary antibodies coupled to Alexa 488 or 568 (Life Technologies). Nuclear staining was performed by adding 1/2000 DAPI (1 mg/mL, Life Technologies) in phosphate-buffered saline for 10 min. Slides were then analyzed with a Zeiss LSM710 confocal laser scanning microscope (60× magnification). Images were collected in the Z direction at 0.80 mm intervals and quantified using the ImageJ plug-in as described previously [14].

### 2.7. Proximity Ligation Assay

Proximity ligation assay was performed with a duolink proximity ligation assay kit (Sigma-Aldrich) according to the manufacturer’s guidelines. 53BP1 (Millipore) and dynein (Abcam, Cambridge, GB) were used to visualize 53BP1-dynein proximity.

### 2.8. DNA Repair Reporter Assays

HeLa cells with stably integrated HR (DR-GFP) or nonhomologous end joining (EJ5GFP) reporters were plated in 6-well plates and then transfected with a plasmid encoding I-SceI endonuclease gene with or without Tau. The cells were left to grow for 48 h and then trypsinized and washed once with PBS. GFP fluorescence was analyzed using an LSR FORTESSA X20 cytometer (Becton Dickinson Biosciences, Haryana, India).

### 2.9. Xenograft Studies

The animals were maintained in compliance with European standards for the care and use of laboratory animals and experimental protocols approved by the local Animal Ethical Committee (agreement APAFIS# 27259-2020091810585608, Lille, France). Ten million MCF7, MCF7 shCtrl, or MCF7 shTau cells were suspended in 100 µL of FBS/Matrigel *v*/*v* (Invitrogen, Carlsbad, CA, USA) and then injected in the fat pad of 6-week-old female NOD/SCID mice (Jackson Laboratory, Bar Harbor, ME, USA). Tumors were measured with calipers, calculated by the formula volume = L × l^2^ × ½. When tumors reached ~100 mm^3^, mice were assigned randomly as either control, doxorubicin (6 mg/kg once via intravenous injection), or X-rays (N = 10 tumors). Mice were then treated with PBS or doxorubicin (6 mg/kg). For X-rays, mice were anesthetized (ketamine, 100 mg/kg and xylazine, 20 mg/kg), and the tumor site was then exposed to radiation (2 Gy × 2) using an XRAD 320. Tumors were measured with calipers, and volume was calculated as described previously.

### 2.10. Statistical Analysis

Data are mean ± SD. Statistical tests were carried out using GraphPad Prism software (GraphPad Inc., La Jolla, CA, USA). The statistical significance between groups was analyzed with the Wilcoxon–Mann–Whitney test and Mann–Whitney tests. A *p*-value less than 0.05 was considered significant.

## 3. Results

### 3.1. Tau Aids Clearance of Double-Strand Breaks

**Figure 1:**
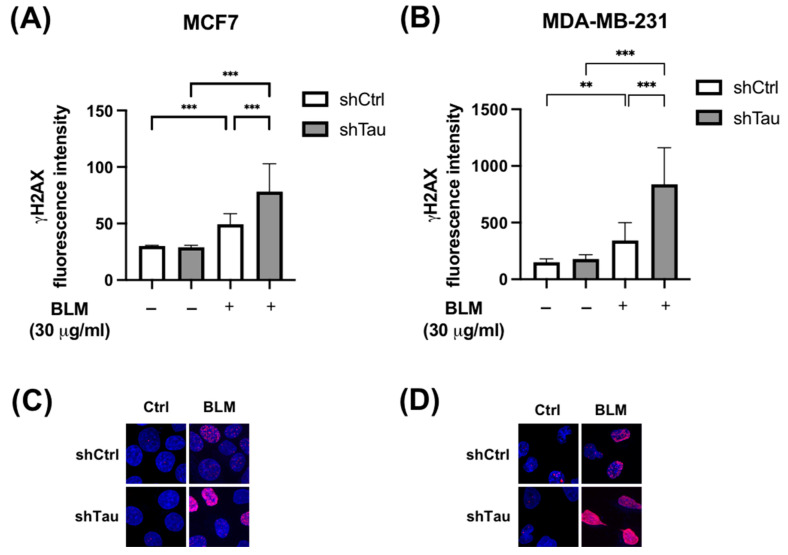
Inhibition of Tau increases γ -H2AX levels after bleomycin treatment in MCF7 and MDA-MB-231. (**A**) MCF7 and (**B**) MDA-MB-231 shCtrl or shTau stable clones were or were not treated with bleomycin (30 μg/mL) for 2 h. Cells were then labeled with a γ-H2AX antibody to quantify DNA double-strand breaks. Nuclear γ-H2AX bulk fluorescence intensity was analyzed using Image J. All experiments were carried out in three independent experiments. Data are mean ± SD (n > 60 cells per conditions) ** *p* < 0.01; *** *p* < 0.001. Representative images of γ-H2AX (red) staining in (**C**) MCF7 and (**D**) MDA-MB-231 shCtrl or shTau stable clones treated or not with bleomycin (30 μg/mL) for 2 h. DAPI nuclear counterstaining is shown in blue.

To assess the potential role of Tau in protecting the DNA of cancer cells, we used MCF7 and MDA-MD-231 cells stably overexpressing either small hairpin RNA control (shCtrl) or targeting Tau (shTau) in which we observed 70% decrease in Tau expression [14]. These control and Tau knockdown cells were treated for 2 h with bleomycin (30 μg/mL), a drug known to induce double-strand breaks. We then quantified DSBs using a phosphorylated form of H2AX as an indicator of DNA damage [29]. Where we knocked down Tau, γ-H2AX fluorescence intensity increased 4-fold compared to just 2-fold in both MCF7 and MDA-MD-231 control cells (Figure 1).

**Figure 2:**
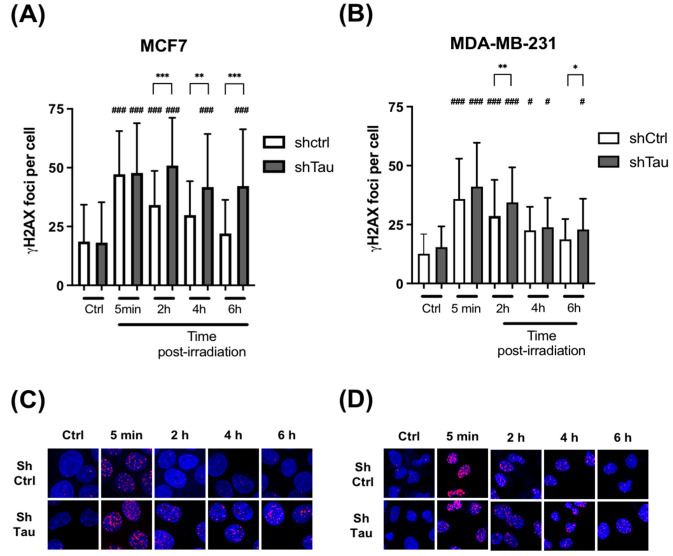
Inhibition of Tau increases γ -H2AX levels after X-ray treatment in MCF7 and MDA-MB-231. (**A**) MCF7 and (**B**) MDA-MB-231 shCtrl or shTau stable clones were X-irradiated with 2 Gy and then further incubated for 5 min, 2, 4, and 6 h. Cells were then labeled with a γ-H2AX antibody to quantify DNA double-strand breaks, and the number of γ-H2AX foci per cell was determined using Image J. All experiments were carried out in three independent experiments. Data are mean ± SD (n > 60 cells per conditions) * *p* < 0.05; ** *p* < 0.01; *** *p* < 0.001. # *p* < 0.05; ### *p* < 0.001 compared to untreated shCtrl cells. Representative images of γ-H2AX (red) staining in (**C**) MCF7 and (**D**) MDA-MB-231 shCtrl or shTau stable clones irradiated or not with 2 Gy. DAPI nuclear counterstaining is shown in blue.

Next, we tested the effect of knocking down Tau on DSB clearance kinetics after a pulse of X irradiation. MCF7 and MDA-MD-231 clones were irradiated with 2 Gy, and the number of γ-H2AX foci was then quantified at 5 min, 2, 4, and 6 h post-irradiation. A 2-fold increase in DSB foci in both MCF7-shCtrl and shTau cell clones was observed. However, while the level of γ-H2AX foci decreased after 2h and had nearly returned to its initial level after 6h in MCF7-shCtrl cells, it remained unchanged 6h post-irradiation in MCF7-shTau cells (Figure 2A,C). We obtained similar results using another shRNA directed against Tau ruling out possible target effects (Supplementary Figure S1). The same trends were observed in MDA-MD-231 shCtrl and shTau cells (Figure 2B,D).

Altogether, these observations suggest a role for Tau in DSB clearance/repair.

### 3.2. Tau Decreases the Mutation Rate Induced by DNA Damaging Agents

**Figure 3:**
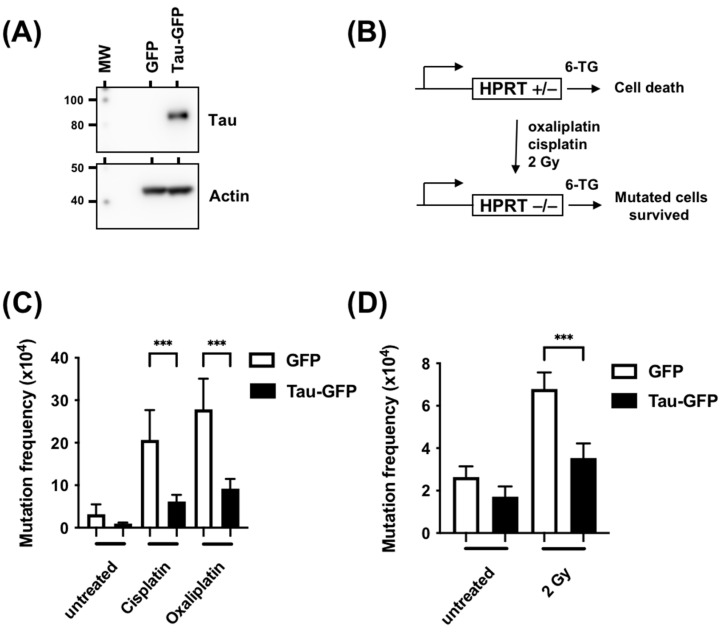
Tau inhibition increases mutation frequency measured by the Hprt test. (**A**) Western blot analysis of Tau expression in CHO-GFP and CHO-Tau-GFP stable clones using anti-Tau and anti-actin antibodies. (**B**) Schematic representation of the HPRT test in CHO cells. (**C**) CHO cells were stably transfected with plasmids coding for GFP or GFP-Tau and treated with cisplatin (10 μM, 2 h) or oxaliplatin (20 μM, 2 h). Mutation frequency was measured as the number of colony/number of cells seeded X efficiency of plating. (**D**) CHO cells were stably transfected with plasmids coding for GFP or GFP-Tau and treated with X-rays (4 Gy). Mutation frequency was determined as described in B. Data are mean ± SD *** *p* < 0.001.

The above results suggested that Tau could be a factor that mediates resistance to DNA-damaging agents. We, therefore, performed a classic Hprt mutation test to assess the rates of spontaneous or cisplatin-, oxaliplatin-, and X-ray-induced mutations in the absence or presence of Tau. CHO K1 cells, which possess only one functional Hprt allele, were stably transfected with plasmids encoding GFP or GFP-Tau fusion proteins, then treated with cisplatin, oxaliplatin, or X-rays (Figure 3A). The addition of 6-thioguanine allowed the selection of Hprt mutants, which reflected the mutation frequencies (Figure 3B). Figure 3C,D demonstrate that the frequency of spontaneous 6-thioguanine-resistant mutants was not significantly different in CHO K1 cells expressing Tau compared to GFP control cells. To study if Tau reduced the frequency of DNA-damaging-agent-induced mutations at the Hprt locus, GFP or GFP-Tau CHO K1 cells were treated with cisplatin (10 μM, 2 h), oxaliplatin (20 μM, 2 h), or X-rays (4 Gy). In the three cases, the frequencies of 6-thioguanine induced by these compounds were significantly lower in cells expressing Tau (3.3-, 3.1-, and 2.8-fold for cisplatin, oxaliplatin, and X-rays, respectively).

Thus, these results confirmed a significant role for Tau in the repair of anticancer-agent-induced DNA lesions.

### 3.3. Tau Increases HR and cNHEJ Activities

**Figure 4:**
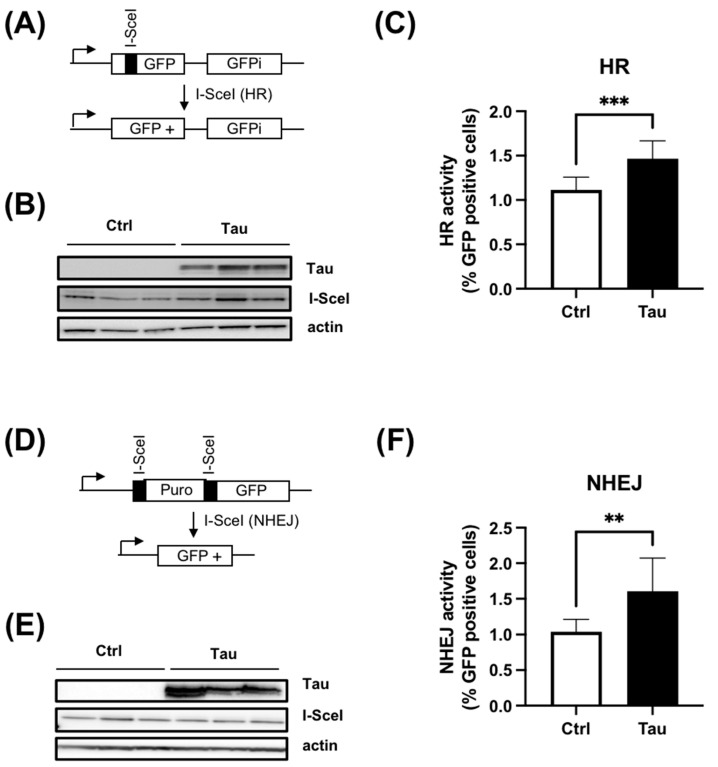
Tau increases HR and cNHEJ activity. (**A**) Schematic representation of DR-GFP reporter. (**B**) Western blot analyses of a HeLa DR-GFP stable clone transiently transfected with plasmid encoding the endonuclease I-SceI with or without plasmid encoding Tau protein. (**C**) Tau increases HR activity. HeLa DR-GFP cells were transfected as described in (**B**). The percentage of GFP-positive cells was quantitated by flow cytometry (five independent experiments). Results are expressed as fold induction relative to the control. (**D**) Schematic representation of EJ5GFP reporter. (**E**) Western blot analyses of HeLa EJ5-GFP stable clone transiently transfected with plasmid encoding the endonuclease I-SceI with or without plasmid encoding Tau protein. (**F**) Tau increased cNHEJ activity. HeLa EJ5-GFP cells were transfected as described in (**E**). The percentage of GFP-positive cells was quantitated by flow cytometry (Five independent experiments). Results are expressed as fold induction relative to the control. Data are mean ± SD ** *p* < 0.01; *** *p* < 0.001.

The repair of DSBs in mammalian cells occurs mainly via the cNHEJ or the HR pathway. To ascertain if Tau acts on a specific DSB pathway, we employed reporter genes of either HR or cNHEJ. Firstly, we employed the HR reporter gene (DR-GFP), which contains a non-functional GFP due to the replacement of 11 bp of the GFP sequence by the rare cutting I-SceI endonuclease recognition site. The I-SceI endonuclease generates a DSB. The HR repair restores the proper GFP coding sequence, resulting in GFP expression, which is further quantified by flow cytometry (Figure 4A) [24]. We established a HeLa DR-GFP stable clone and then transiently transfected a plasmid encoding the I-SceI endonuclease in the absence (Ctrl) or presence of plasmid encoding Tau (Tau). The endonuclease expression level was verified and shown to be similar between HeLa-DR-GFP-Ctrl and HeLa-DR-GFP-Tau cells (Figure 4B). Noticeably, Tau overexpression resulted in a 1.5-fold increase in the number of cells expressing GFP in HeLa-DR-GFP-Tau (Figure 4C). These results indicated that Tau promoted more efficient HR repair.

Secondly, we evaluated the cNHEJ DNA repair process using the EJ5-GFP reporter [23]. In this plasmid, the promoter is separated from the GFP by a puromycin gene containing two I-SceI sites at the 5′ and 3′ positions. Following I-SceI cleavage, the puromycin gene is excised, leading to the restoration of the GFP signal by NHEJ repair (Figure 4D). A selected HeLa EJ5-GFP clone was therefore transfected with a plasmid encoding the I-SceI in the absence or presence of plasmid encoding Tau. As above, the level of I-SceI expression was similar between the two conditions (Figure 4E). As for HR, we observed a 1.6-fold increase in cNHEJ repair activity when Tau was expressed (Figure 4F).

Taken together, these data demonstrated that Tau increased both HR and cNHEJ repair efficiency.

### 3.4. shTau Tumors Are More Sensitive to DNA Damage

**Figure 5:**
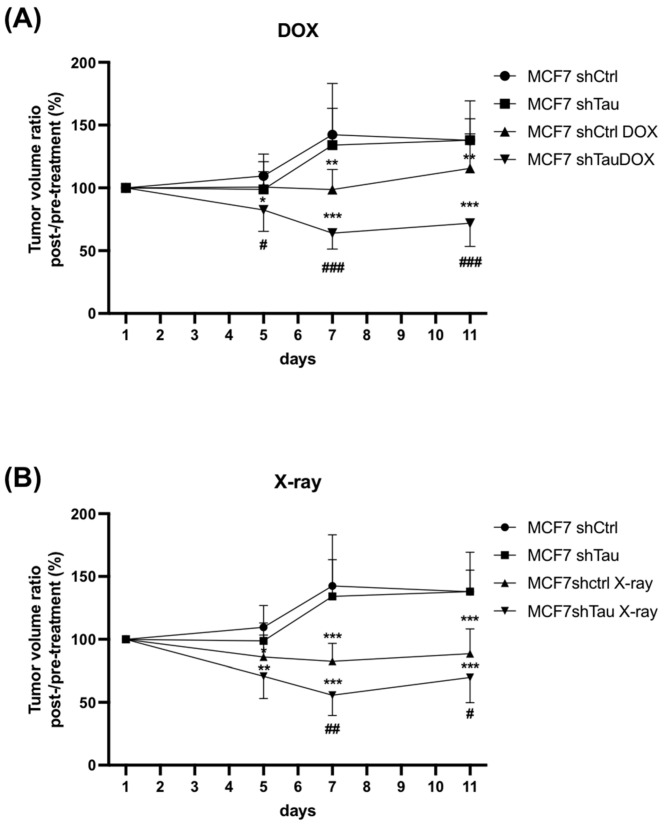
Tau depletion confers sensitivity to doxorubicin and X-rays in mice xenografts. MCF7 cells expressing either scramble (shctrl) or shTau were injected subcutaneously (5 mice per group), and tumor volume was measured for 11 days starting from 100 mm^3^ and (**A**) treated or not with doxorubicin (6 mg/kg × 1) or (**B**) treated or not with X-rays (2 Gy × 2). Average tumor weight ± SD is shown. * *p* < 0.05; ** *p* < 0.01; *** *p* < 0.001 compared to untreated cells. # *p* < 0.05; ## *p* < 0.01 ### *p* < 0.001 compared to treated MCF7 shCtrl xenografts.

Finally, we sought to test the in vivo relevance of our cellular findings. For this, MCF7-shCtrl and shTau cells were injected subcutaneously into the mammary fat pads of immunodeficient SCID mice. The tumor volumes were measured every three days until they reached 100 mm^3^ and then treated with X-rays or the widely used chemotherapy drug, doxorubicin. As shown in Figure 5A, there were no differences in tumor growth between shCtrl and shTau xenografts during these eleven days in the absence of doxorubicin. When treated with doxorubicin, shCtrl xenografts remained at approximately the starting volume, neither growing nor shrinking. For shTau xenografts, however, doxorubicin treatment led to a decrease in tumor volume of 12.7% and 28.1% on days five and eleven, respectively (Figure 5A). Similar results were observed after X-ray treatment, with X-rays reducing tumor volume more in the shTau xenografts, with a 29.2% and 30.2% decrease on days five and eleven, respectively (Figure 5B).

Together, these data strongly support that Tau inhibition potentiates the therapeutic effect of doxorubicin and X-rays by inhibiting DNA repair.

### 3.5. Tau Regulates 53BP1 Nuclear Localization

While we observed that Tau increased both cNHEJ and HR, it is known that cNHEJ accounts for most DSB repair in mammalian cells [7,8]. In addition, the recruitment of 53BP1 at DSBs is known to be critical for this mechanism [6].

**Figure 6:**
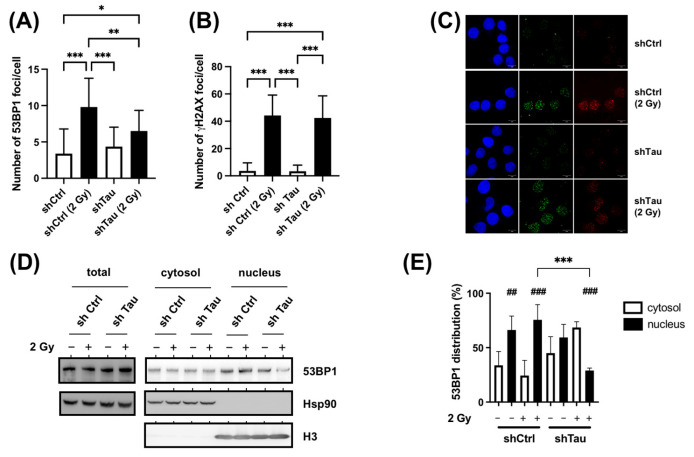
Tau increases 53BP1 nuclear translocation after X-ray treatment. (**A**). MCF7 shCtrl and shTau cells were irradiated with 2 Gy exposure and then further incubated for 15 min. Cells were then labeled with a 53BP1 antibody, and the number of 53BP1 foci per cell (n > 50) was determined using Image J. All experiments were carried out in three independent experiments. Data are mean ± SD * *p* < 0.05; ** *p* < 0.01; *** *p* < 0.001. (**B**) MCF7 shCtrl or shTau stable clones were irradiated with 2 Gy and then further incubated for 15 min. Cells were then labeled with a γ-H2AX antibody to quantify DNA double-strand breaks, and the number of γ-H2AX foci per cell (n >50) was determined using Image J. All experiments were carried out three independent experiments. Data are mean ± SD *** *p* < 0.001. (**C**) Representative images of 53BP1 (red) and γ-H2AX (green) staining in MCF7 shCtrl or shTau stable clones irradiated or not with 2 Gy and further incubated for 15 min. (**D**) Total extraction and subcellular fractionation of MCF7 shCtrl and shTau cells 15 min after X-ray treatment was followed by western blots to monitor the levels of 53BP1 in the cytoplasm and nucleus. HSP90 was used as a marker of total and cytoplasmic fractions, and histone H3 as a marker of the nuclear fraction. (**E**) The graph shows the quantification of four independent experiments from C. Data are mean ± SD * *p* < 0.05; ** *p* < 0.01; *** *p* < 0.001. ## *p* < 0.01; ### *p* < 0.001 compared to the corresponding cytoplasmic fraction.

We next examined the formation of 53BP1 foci in MCF7 shCtrl and shTau cells by immunofluorescence. As shown in Figure 6A and C, we observed a 4.5-fold increase in the number of 53BP1 foci in shCtrl cells 15 min after 2 Gy irradiation. However, knocking down Tau expression led to a decrease in 53BP1 foci number (2.1-fold increase 15 min after 2 Gy irradiation). On the other hand, Tau knockdown had no effect on the number of γ-H2AX foci (Figure 6B,C). Similar results were found 30 min after 2 Gy irradiation.

The lower abundance of nuclear 53BP1 foci was not explained by an expression change (Figure 6D, Total). We, therefore, hypothesized that the observed differences in shCtrl and shTau conditions could be due to a perturbed nuclear localization. Indeed, it has been demonstrated that efficient DSB repair relies on the nuclear translocation of DNA repair proteins such as 53BP1 [9]. To test this, intracellular 53BP1 distribution was tested using subcellular fractionation (Figure 6D,E). For MCF7 shCtrl cells, 53BP1 protein was found primarily in the nuclear fraction in control (66%) and irradiated conditions (75%). Although the distribution was similar in the MCF7 shTau cells in control conditions (60% in the nuclear fraction), we observed a strong decrease in the nuclear fraction after X-ray treatment (30%).

These data, therefore, suggest that Tau regulates the cytonuclear shuttling of 53BP1.

### 3.6. Tau Silencing Alters 53BP1 Trafficking on Microtubules

**Figure 7:**
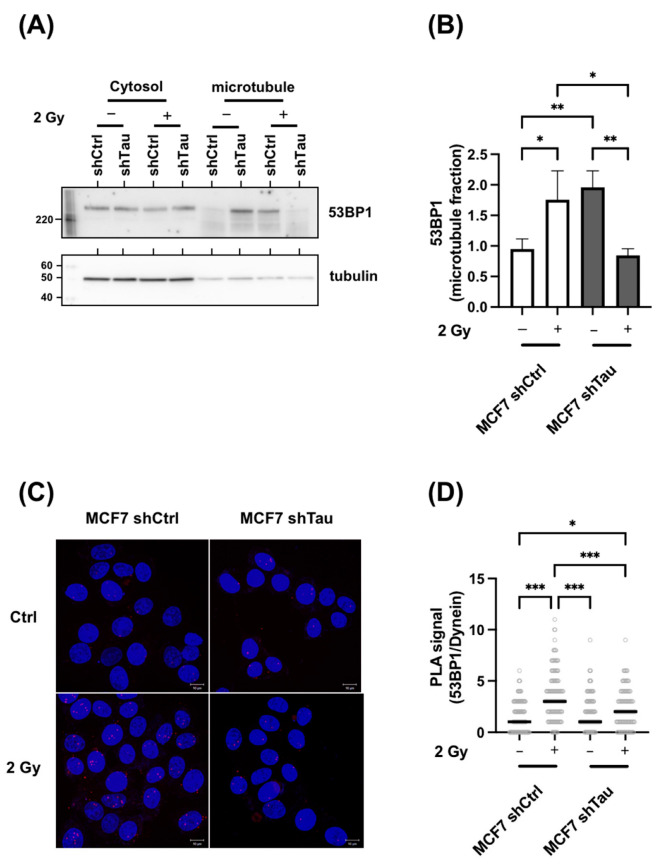
Tau regulates the 53BP1/microtubule interaction. (**A**) Representative 53BP1 and tubulin western blot analysis of microtubule fractions from MCF7 shCtrl and shTau cells in control or 2 Gy conditions. (**B**) The graph shows the quantification of three independent microtubule fractionation experiments followed by 53BP1 and tubulin detection by western blot. (**C**) Representative in situ Proximity Ligation Assay showing 53BP1-Dynein interaction in MCF7shCtrl and shTau cells in control and 2 Gy treated conditions. The maximum intensity projection (MIP) of the z-stack is shown. (**D**) The graph shows the quantification of three independent proximity ligation assays (53BP1-Dynein) experiments as in C. All data are mean ± SD * *p* < 0.05; ** *p* < 0.01; *** *p* < 0.001.

It has been shown that the nuclear translocation of 53BP1 and other DNA repair proteins relies in part on their interaction with the retrograde motor protein dynein [9]. Since Tau is mostly known as a microtubule-binding protein that regulates dynein, we next wondered if its absence could impede the interaction of 53BP1 with microtubules. We first compared the microtubule association of 53BP1. For this, 53BP1 distribution between the cytosolic and microtubule fraction was assessed in control or X-ray-treated MCF7-shCtrl or -shTau cells. As shown in Figure 7A,B, 53BP1 is present in both cytoplasmic and microtubule fractions. Noticeably, we observed a 2-fold difference in 53BP1-microtubule complex enrichment in non-treated MCF7-shTau cells when compared to non-treated MCF7-shCtrl. Moreover, we found that 53BP1-microtubule association was further increased in MCF7shCtrl cells after 2 Gy irradiation (1.9-fold increase) while decreased in MCF7 shTau cells (2.1-fold decrease).

Since the higher association could indicate either an increased or a stronger association of 53BP1 with microtubules, we next examined whether there were also differences in 53BP1/dynein interaction. For this, a proximity ligation assay was performed to specifically detect 53BP1/dynein interaction in MCF7-shCtrl and shTau cells in control or 2 Gy treated conditions. As shown in Figure 7C,D, proximity was increased in shCtrl cells after irradiation (2.3-fold), while the signal was lowered in MCF7-shTau cells (1.2-fold increase).

## 4. Discussion

In this work, we investigated the role of Tau in repairing DNA double-strand breaks in cancer cells. We reported a new function of Tau in DSB repair that it carries out by regulating DNA repair protein trafficking. Notably, we identified Tau as a major actor in regulating cNHEJ through regulating 53BP1 trafficking on microtubules.

These findings have precedents in several studies, suggesting a relationship between Tau and DNA damage [15,30]. In particular, an increase in γ-H2AX foci was observed under stress conditions in primary neuronal cell cultures in the absence of Tau or in the cortex and hippocampus of knock-out Tau mice [31,32]. Although it has been suggested that Tau binds and protects DNA from damage, our observations do not support this hypothesis. First, the same level of H2AX phosphorylation was observed in shCtrl and shTau cells after irradiation, which seems to be incompatible with a direct DNA protective role for Tau. Second, we observed a delay in the resolution of γ-H2AX foci in shTau cells that further suggested a role for Tau in DNA repair as opposed to DNA binding per se.

The observed kinetics of γ-H2AX resolution suggested that Tau mostly assists DNA repair through the cNHEJ pathway. To obtain more detailed insights into Tau function in cNHEJ, we examined the formation of 53BP1 foci in shCtrl and shTau cells. A lower 53BP1 recruitment to DNA damage sites is known to be sufficient to hinder DSB repair by cNHEJ [33]. However, while previous studies report a decrease in 53BP1 expression upon degradation, we did not observe variations in protein level but rather an alteration in protein localization. Dysregulation of nucleocytoplasmic transport has previously been implicated in neuronal Tau pathology. Protein aggregates have been shown to interact with different components of the nuclear pore complex and alter nucleocytoplasmic transport [34,35]. Using Frontotemporal Lobar Degeneration Tau mutations (FTDP-MAPT), others demonstrated that changes in microtubule dynamics caused nuclear envelope distortions and marked disruption of nucleocytoplasmic transport [36]. These observations suggest that Tau loss-of-function impedes microtubule nuclear translocation.

Our results, therefore, suggest that 53BP1 nucleocytoplasmic shuttling is highly dependent on Tau. 53BP1 is present both in the cytoplasm and nucleus in shCtrl cells, and the nuclear translocation of 53BP1 tends to increase after DSB induction. Furthermore, we observed increased interaction of 53BP1 with both dynein and microtubules in the presence of Tau when treated with DNA-damaging agents. Interestingly, it was observed that enhanced nuclear targeting of the human adenovirus type 2 (Ad2) after overexpression of MAP4 was reliant on directed transport along microtubules by dynein [37]. These observations further suggest that Tau might exert a similar role in dynein-directed protein translocation. Indeed, Tau depletion led to strong alterations in dynein-directed protein translocation. In shTau control cells, we observed an increase in the 53BP1 microtubule fraction independent of nuclear translocation. Although the current explanation is not known, this might reflect a lower displacement rate of the dynein complex along microtubules. Tau is known to increase the frequency of long dynein-directed runs [38]. Altogether, these observations are in agreement with previous results showing that altering microtubule dynamics with vincristine and paclitaxel increased 53BP1 cytoplasmic retention and accentuated the effect of DNA-damaging agents [9].

It is important to note that an alteration in 53BP1 protein localization is observed after DSB induction, suggesting that microtubule dynamics are regulated by DSBs themselves. It has been demonstrated that upon double-strand breakage, cytoplasmic microtubules invade the nucleus [39]. This mechanism is thought to increase the mobility of damaged DNA inside the nucleus necessary for DNA repair [10,40]. Interestingly, it has been shown recently in neurons that DSBs increased the interaction between Tau and tubulin around the nuclear membrane, further suggesting the enhancement of tubulin polymerization [32]. Based on our observations, it is tempting to speculate that Tau responds to DSBs by enhancing microtubule polymerization and/or the stability necessary for DNA mobility and nuclear translocation of DNA repair proteins.

Transport of DNA repair proteins along microtubules is not restricted to 53BP1 but also includes other proteins involved in different DNA repair pathways. P53, DNA-PK complex proteins (Ku70, Ku80, DNA-PKcs), BRCA1/2, MRN complex proteins (Mre11, Rad50, Nbs1), or RPA and Rad51 are also subjected to microtubule trafficking [9,41,42,43]. In this regard, we found that the efficiency of the HR pathway in reporter gene assays is increased in the presence of Tau. This observation further suggests that Tau did not impact the DSB repair pathway choice. In this regard, we also show that Tau-expressing cells are less sensitive to cisplatin and oxaliplatin treatments. Platinum drugs act mainly by forming intrastrand diadducts, repaired via the nucleotide excision repair system [44]. Interestingly, cytosolic sequestration of DNA repair proteins has been linked to neuronal death in different Tauopathies such as Alzheimer’s disease, Pick’s disease, corticobasal neurodegeneration, or progressive supranuclear palsy [45,46].

## 5. Conclusions

In this work, we provide evidence for a new role of Tau in DNA repair, promoting resistance to commonly used anti-cancer treatments. These findings further suggest that Tau expression may be of interest as a molecular marker for response to DNA-damaging agents and as a beneficial therapeutic target in tumors.

## Data Availability

All materials are fully available on request.

## Supplementary Materials

The following supporting information can be downloaded at: https://www.mdpi.com/article/10.3390/cancers15010116/s1. Supplementary Figure S1: Inhibition of Tau increases γ-H2AX levels after X-ray treatments in MCF7. File S1: Original blots.
