# Supplementary material for: Cancer Cells Upregulate Tau to Gain Resistance to DNA Damaging Agents
Source: Cancers (Basel). 2022 Dec 24;15(1):116. doi: 10.3390/cancers15010116 (PMC9817522; doi:10.3390/cancers15010116)
Supplement: Supplementary file 1 [file cancers-15-00116-s001.zip › cancers-2082718-File S1.pdf]

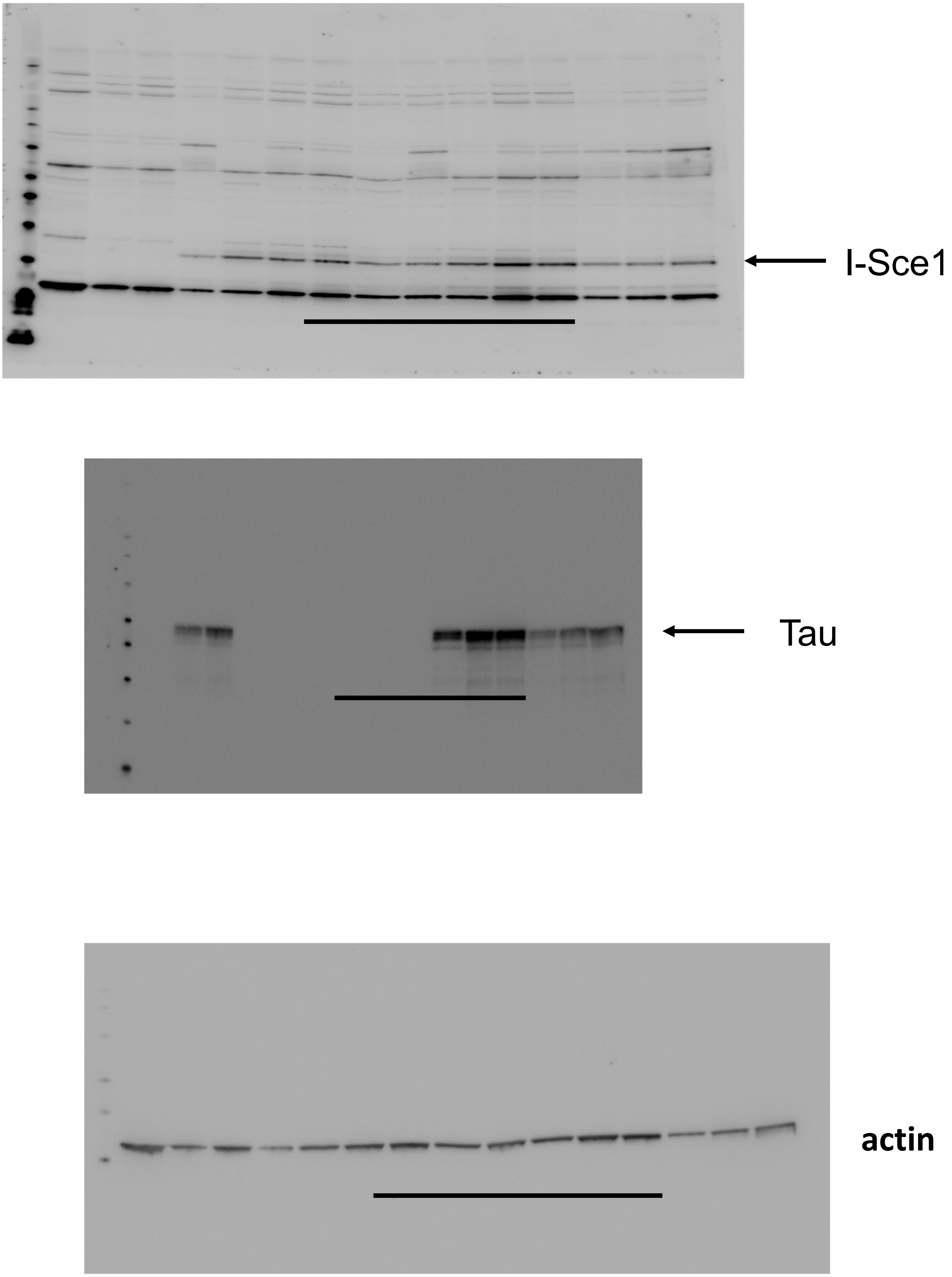

FIGURE 3B

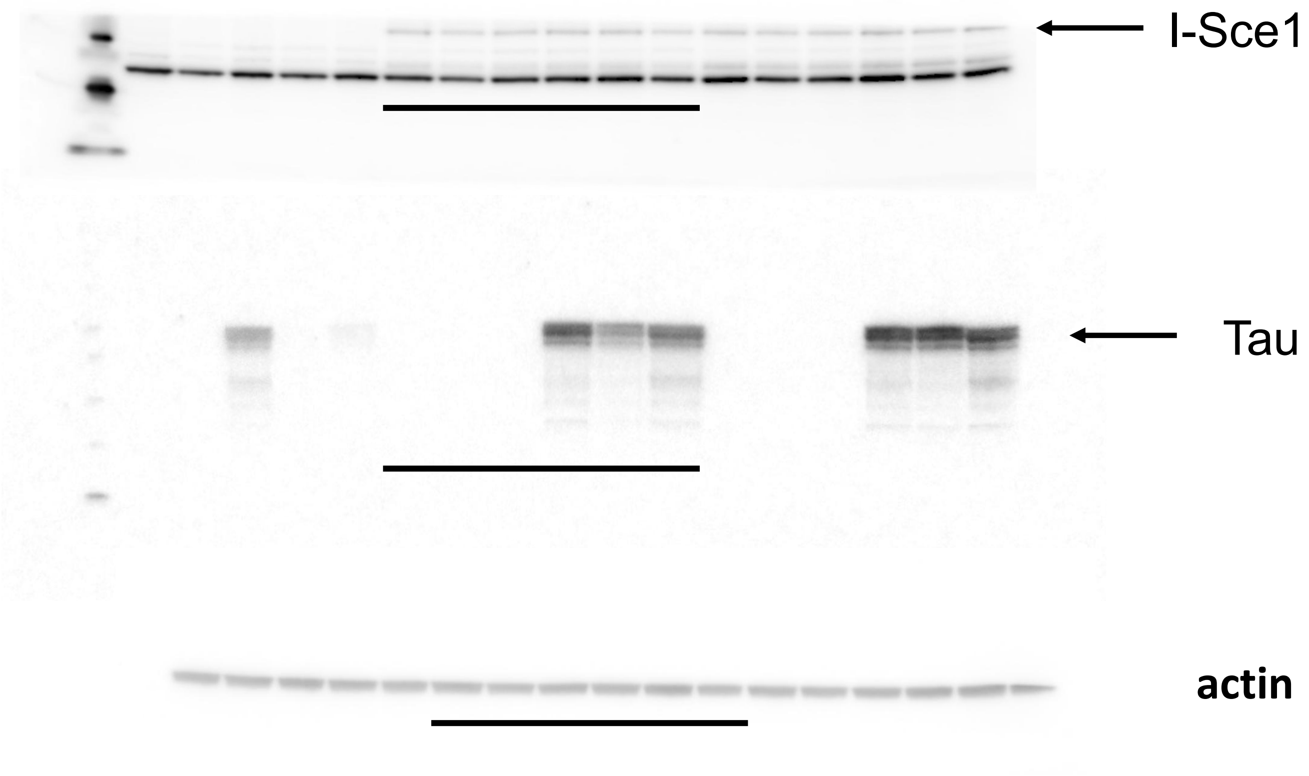

FIGURE 3E

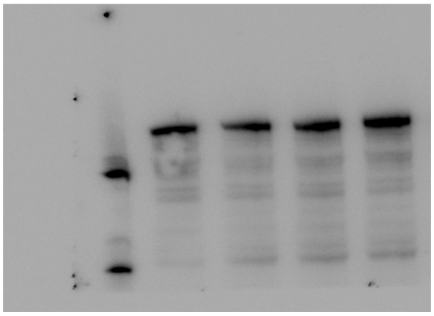

53BP1
(total extraction)

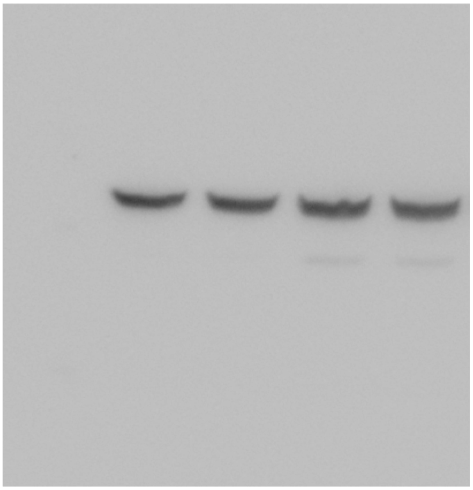

Hsp90
(total extraction)

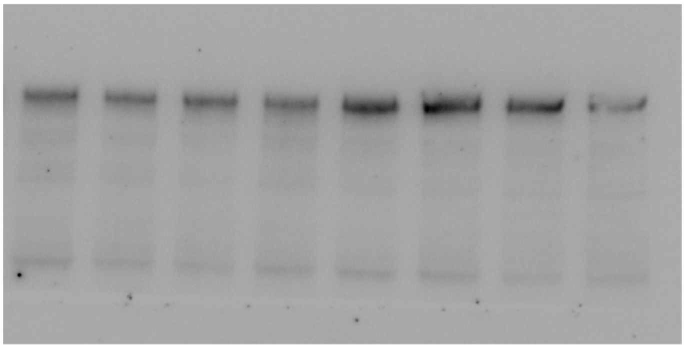

53BP1
(fractionation)

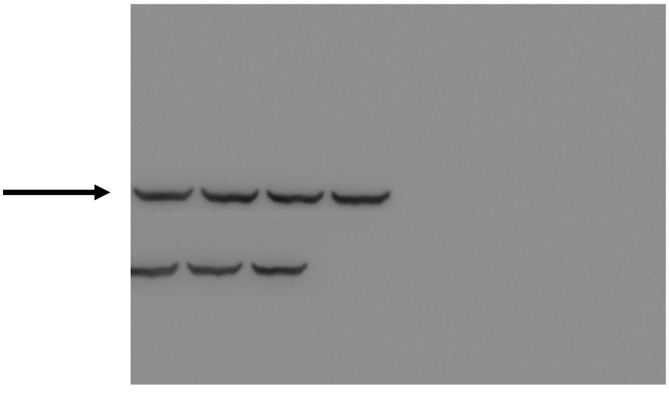

Hsp90
(fractionation)

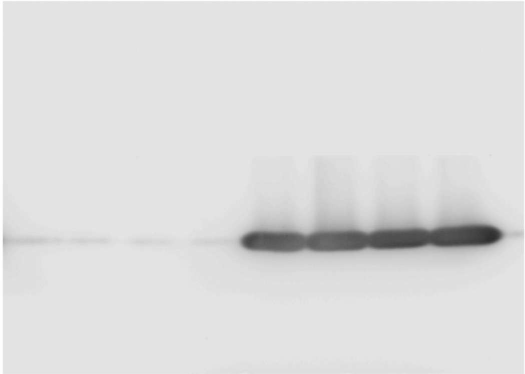

H3
(fractionation)

FIGURE 5C

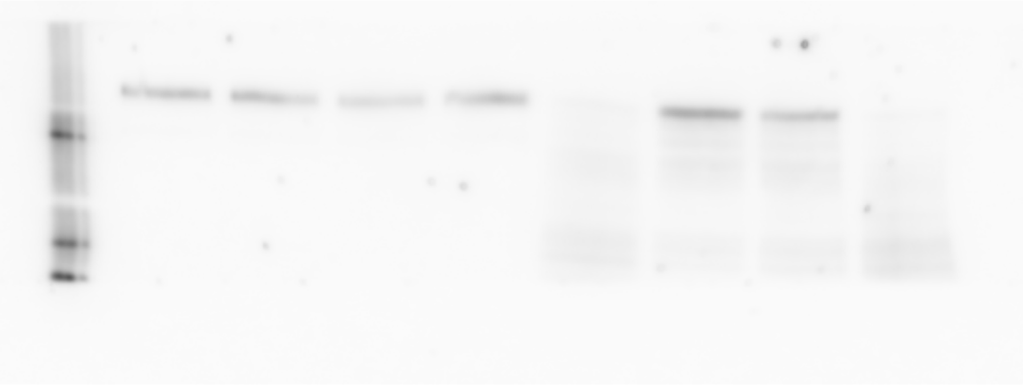

53BP1

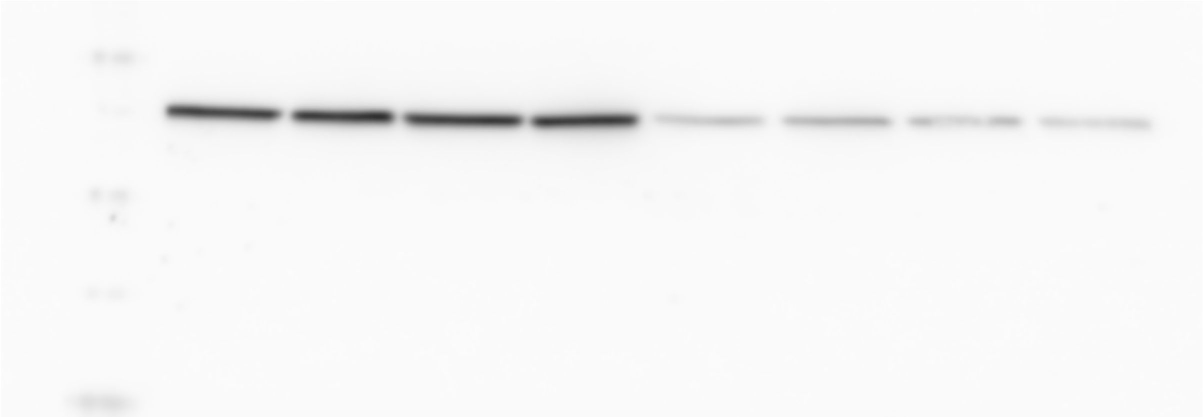

Tubulin

FIGURE 6A
